# Impact of adverse childhood experiences on health-related quality of life in Australian women with endometriosis: a population-based cohort study

**DOI:** 10.1007/s11136-025-04160-1

**Published:** 2026-02-01

**Authors:** Dereje G. Gete, Jenny Doust, Sally Mortlock, Jason Abbott, Gita D. Mishra

**Affiliations:** 1 Australian Women and Girls’ Health Research Centre, School of Public Health, The University of Queensland, Brisbane, QLD 4006 Australia; 2 School of Clinical Medicine, Health and Medicine, The University of New South Wales, Sydney, NSW 2052 Australia

**Keywords:** Health-related quality of life, Adverse childhood experiences, Endometriosis, Endometriosis health profile-30

## Abstract

**Purpose:**

To examine the associations between adverse childhood experiences (ACEs) and health-related quality of life (HRQoL) among Australian women with endometriosis.

**Methods:**

This population-based cohort study included 1,120 women with endometriosis born in 1973–78 and 1989–95, using data from the Australian Longitudinal Study on Women’s Health and its sub-study, the Genetic variants, Early Life exposures, and Longitudinal Endometriosis symptoms Study. HRQoL was assessed using the Endometriosis Health Profile-30, and ACEs were measured using a validated 21-item questionnaire. Ordinal logistic regression models were used for analyses.

**Results:**

Women who experienced ACEs were more likely to be diagnosed with endometriosis than those who did not. Among women with endometriosis, a clear dose-response relationship was observed, with higher cumulative ACE scores and greater trauma severity associated with progressively poorer HRQoL across all domains. In the 1973–78 cohort, the strongest associations between cumulative ACEs and HRQoL were observed in the domains of social support, self-image, and emotional well-being, with adjusted odds ratios (95% CIs) of 1.41 (1.24, 1.58), 1.41 (1.24, 1.60), and 1.30 (1.15, 1.46), respectively. Women reporting very/extremely traumatic experiences also had significantly worse outcomes across all domains, particularly for social support 4.42 (2.36, 8.28), emotional well-being 4.38 (2.36, 8.12), and self-image 3.29 (1.77, 6.11). Psychological abuse, physical abuse, and household mental illness were consistently associated with poorer HRQoL, particularly in the social support domain. Similar patterns were observed in the 1989–95 cohort, though with some domain-specific variations.

**Conclusion:**

Childhood adversity significantly impairs HRQoL in women with endometriosis, particularly psychological abuse, physical abuse, and household mental illness. These findings highlight the need for trauma-informed care in endometriosis management, with careful consideration of the limitations of routine ACE screening.

**Supplementary Information:**

The online version contains supplementary material available at 10.1007/s11136-025-04160-1.

## Introduction

Adverse childhood experiences (ACEs) are traumatic or highly stressful events occurring before the age of 18, often leading to lasting impacts on health and well-being [1]. Over 62% of Australians experienced at least one type of childhood maltreatment [2], including high proportions of physical (32%), sexual (28%), and emotional abuse (31%), as well as exposure to domestic violence (40%) [3]. Child maltreatment varies across social groups, with a higher prevalence of sexual and emotional abuse among women and greater multi-type maltreatment among Aboriginal and Torres Strait Islander peoples [4]. ACEs have been strongly associated with increased risks of mental health disorders [5], engagement in health-risk behaviours, and chronic conditions such as cardiovascular disease, diabetes, and obesity [6].

Emerging evidence suggests that ACEs may also contribute to an increased risk of developing endometriosis [7, 8]. Endometriosis is a chronic inflammatory condition characterised by a range of debilitating symptoms that significantly impair health-related quality of life (HRQoL) [9, 10]. Additionally, ACEs have been linked to poorer HRQoL in the general population [11], as well as in specific groups, including cancer survivors [12], youth with chronic pain [13], and women undergoing hysterectomy for uterine leiomyoma [14]. However, to our knowledge, no study has yet examined the impact of ACEs on HRQoL in women with endometriosis. Investigating this association is essential for identifying vulnerable populations, improving clinical management, and developing targeted interventions to enhance long-term health outcomes.

HRQoL encompasses physical, mental, and social well-being in the context of disease or treatment [15]. While generic tools like the 36-item short-form health survey are widely used, they may not fully capture the specific impact of endometriosis [16, 17]. To address this limitation, the 30-item endometriosis health profile (EHP-30) was developed in 2001 through patient interviews [18] and is now the most validated and reliable instrument for assessing HRQoL in women with endometriosis [19].

This study aims to examine the impact of ACEs on HRQoL, as measured by the EHP-30, in Australian women of reproductive age diagnosed with endometriosis. The findings may offer valuable insights into the long-term impact of early-life adversity on women’s health and inform strategies for more comprehensive, patient-centred care.

## Methods

### Study design and populations

This study utilised data from the Australian Longitudinal Study on Women’s Health (ALSWH) and its sub-study, the Genetic variants, Early Life exposures, and Longitudinal Endometriosis symptoms Study (GELLES). The ALSWH is an ongoing, large-scale prospective cohort study that examines various factors affecting women’s health and well-being. Established in 1996, the study enrolled over 40,000 women from three birth cohorts (born in 1973–78, 1946–51, and 1921–26), randomly selected from Australia’s universal health insurance database (Medicare). From 1996 to 2021, nine surveys were administered online or as postal questionnaires every three years. Detailed descriptions of the study’s design, recruitment strategies, and response rates have been published previously [20, 21].

In 2013, a new cohort of 17,010 women born in 1989-95 was recruited, largely through social media, and participated in six annual surveys from 2013 to 2019 [22, 23]. Women from this cohort were invited to join the GELLES study in November 2021, while those from the 1973–78 cohort received invitations in April 2023. Participants completed an online survey covering early life exposures, reproductive history, and endometriosis diagnoses.

**Fig. 1:**
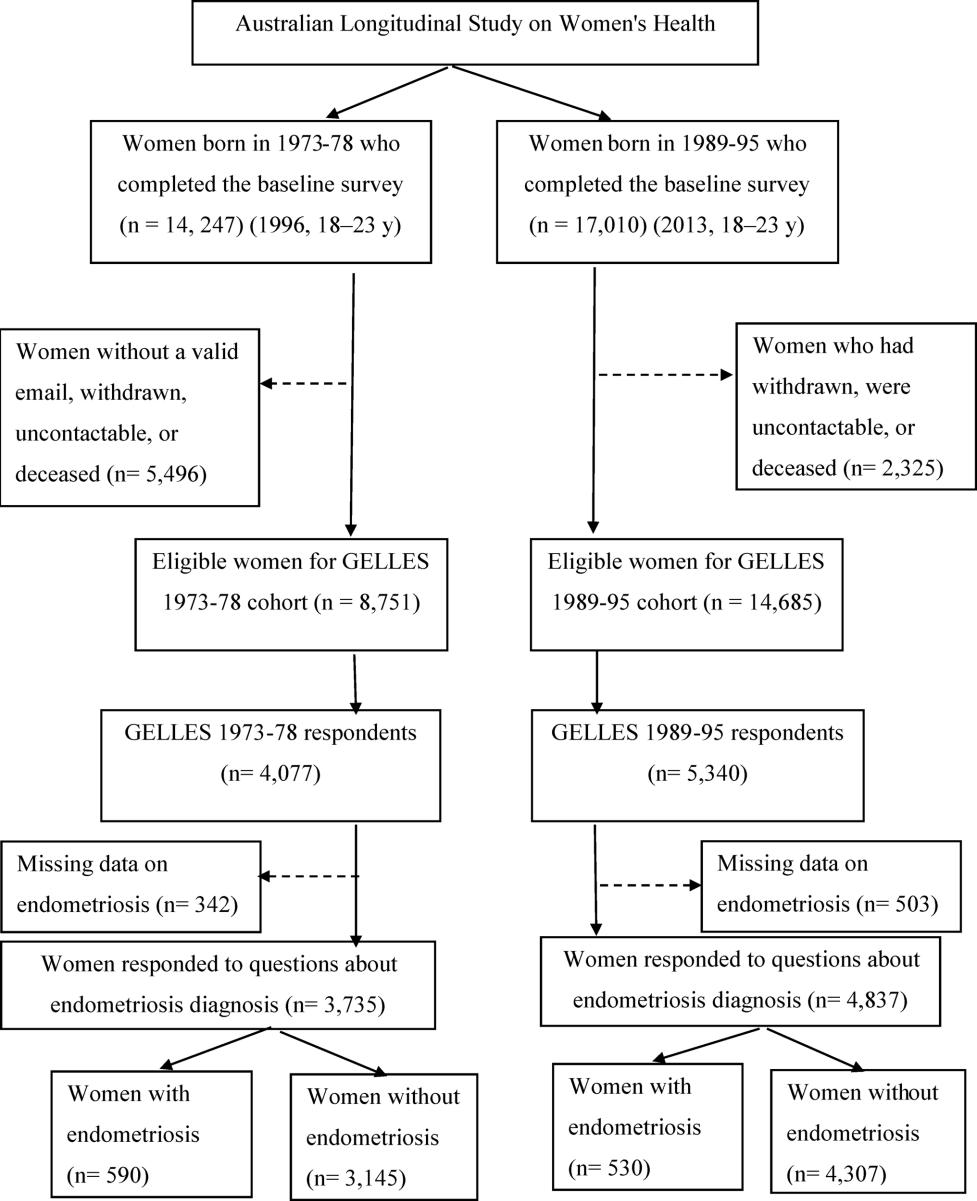
Flowchart of sample selection for the analysis of adverse childhood experiences and health-related quality of life

The current study analysed data from the ALSWH cohorts born in 1973–78 and 1989–95. Women were eligible for the GELLES study if they belonged to either cohort, except those who had withdrawn from ALSWH, did not have a valid email address, were uncontactable, or had passed away. In the 1973–78 cohort, 8,751 women met the eligibility criteria for GELLES, with 4,077 (47%) responding. Among them, 3,735 provided information on endometriosis diagnosis, and 590 (14.5%) reported having the condition. In the 1989–95 cohort, 14,685 women were eligible, and 5,340 (36%) participated. Of the 4,837 who answered questions about endometriosis diagnosis, 530 (9.9%) indicated they had been diagnosed with the condition (Fig. 1).

### Assessment of endometriosis

We identified women with endometriosis using self-reported data from the GELLES survey. Participants were asked, “Has a doctor or other healthcare provider ever diagnosed you with endometriosis?” Those who answered yes were further asked to specify the diagnostic method, including laparoscopy or other surgeries, MRI/ultrasound, or diagnosis based on symptoms. These self-reported diagnoses were validated against ALSWH and linked health records, which were considered the gold standard and showed strong agreement [24].

### Assessments of adverse childhood experiences

Data on ACEs were assessed using self-reported information from the ALSWH cohorts born in 1973–78 and 1989–95. The initial data collection was conducted in 2015, with the 1989–95 cohort reporting at Survey 3 (ages 20–25) and the 1973–78 cohort at Survey 7 (ages 37–42). The ACEs study, first published by (1998) [25], has demonstrated strong reliability, with retrospective reports of childhood adversity remaining stable over time [26]. In the ALSWH survey, participants answered 21 questions about ACEs before age 18, expanding on the original 17 questions [25] with four additional items on witnessing violence against a father or stepfather. These asked whether a father or stepfather was pushed, grabbed, slapped, or had something thrown at him; kicked, bitten, or hit with a fist or hard object; repeatedly hit over several minutes; or threatened or injured with a knife or gun [27]. The questionnaire covered seven domains of childhood abuse and household dysfunction: psychological abuse (2 items), physical abuse (2 items), sexual abuse (4 items), household substance abuse (2 items), household mental illness (2 items), violent treatment of a parent or stepparent (8 items), and household criminal behaviour (1 item). Participants were classified as exposed to a category if they answered “yes” to at least one question within that domain. The ACE score was calculated as the total number of categories in which a participant reported exposure, ranging from 0 (no exposure) to 7 (exposure across all categories).

In the GELLES survey, participants were also asked to reflect on their childhood trauma by responding to the question: “Thinking about your childhood, would you say it was traumatic?” Response options included: not at all, somewhat, moderately, very, and extremely.

### Assessments of health-related quality of life

The GELLES survey assessed women’s HRQoL using the EHP-30, a self-reported, disease-specific questionnaire with 30 items across five subscales, reflecting key challenges faced by women with endometriosis. These subscales include pain (11 items), control & powerlessness (6 items), emotional well-being (6 items), social support (4 items), and self-image (3 items). Participants who reported an endometriosis diagnosis were asked to reflect on their worst endometriosis episode and indicate how frequently, due to their condition, they have experienced specific issues, using a five-point Likert scale ranging from never to always (0–4). Each subscale is then converted into a score from 0 (indicating the best possible HRQoL) to 100 (indicating the worst HRQoL), calculated by dividing the raw score for each subscale by the maximum possible score for that subscale and multiplying by 100 [18].

In this study, the EHP-30 subscale data were skewed. Therefore, each subscale was categorised into tertiles according to its distribution within the sample for analysis.

### Assessments of confounders

Potential confounders were selected based on prior evidence of their associations with both ACEs and HRQoL, and their relevance was further assessed among our study participants. Adjustments were made for parental socio-demographic and economic factors during childhood, including area of residence, parental education, family income management, and parental divorce or separation. Data on these confounders were obtained from the GELLES survey for the 1973–78 cohort. However, the 1989–95 cohort, the GELLES survey, provided information only on the area of residence, with other confounders missing. As a result, the necessary data for this cohort were obtained from the ALSWH Survey 3 (2015).

Parental residence was classified as urban or rural/remote [28]. Parental education was categorised as up to year 12 or equivalent, trade/apprenticeship/certificate/diploma, or university/higher degree. Family income management was assessed based on self-reported financial difficulty: impossible or difficult all the time, difficult sometimes, not too bad, and easy.

### Statistical analysis

Childhood adversity based on women with and without endometriosis was summarised as percentages (%) for each form of ACE and as the median with quartiles for the cumulative ACEs score, using Pearson’s chi-square and Mann-Whitney tests. The EHP-30 domain scores by each form of ACE and level of trauma severity were presented as medians with quartiles, using the Mann-Whitney and Kruskal-Wallis tests.

Data normality and skewness were assessed using a histogram and the Kolmogorov–Smirnov test. The distributional assumptions for linear regression outcomes and residuals were unmet, and the EHP-30 subscale data remained skewed even after log transformation. Consequently, each EHP-30 subscale was further categorised into tertiles based on the sample distribution. Ordinal logistic regression was used to examine the associations of cumulative ACEs, specific ACE types, and trauma severity with HRQoL, with results presented as odds ratios (ORs) and 95% confidence intervals (CIs). This model estimates the cumulative odds of being in a higher tertile of the EHP-30 subscales (indicating worse HRQoL) compared with lower tertiles. The analyses were adjusted for potential confounders, including area of residence, parental education, family income, and parental divorce or separation. Restricted cubic spline analyses showed no evidence of non-linearity or threshold effects; therefore, the ACE score was modelled as a continuous variable. A complete case analysis was the primary approach. To address missing data in exposures (1.3%–13.7%) and confounders (1.3%–24%), and to test the robustness of the results, multiple imputation using chained equations (20 imputations) was performed under the missing-at-random assumption, with findings compared against those from the complete case analysis. Multicollinearity was evaluated using variance inflation factors (VIF) and tolerance. Statistical analyses were performed using Stata software version 18 (StataCorp, College Station, TX) and SAS software version 9.4 (SAS Institute Inc., Cary, NC).

## Results

The first set of analyses compared 7452 women without endometriosis with 1120 women with endometriosis from two Australian birth cohorts, born in 1989–95 and 1973–78 (Fig. 1). In the 1973–78 cohort, 590 women (14.5%) were diagnosed with endometriosis, of whom 483 (11.9%) had a confirmed diagnosis through laparoscopy or other surgical methods, while 107 (2.6%) were diagnosed clinically based on symptoms, MRI, or ultrasound. In the 1989–95 cohort, 530 women (9.9%) reported being diagnosed with endometriosis, of whom 385 (7.2%) were diagnosed through surgical procedures and 145 (2.7%) were diagnosed clinically. The mean age at diagnosis of endometriosis was 31 years (SD 9.4) for the 1973–78 cohort and 24 years (SD 4.6) for the 1989–95 cohort.

Table 1 compares each form of ACE between women with and without endometriosis. Women who experienced ACEs were more likely to be diagnosed with endometriosis than those who did not. In both cohorts, notable differences were observed in the percentage of overall ACE domains between women with and without endometriosis, except for household criminal behaviour, which had a low overall occurrence. Around 49% and 70% of women with endometriosis in the 1973–78 and 1989–95 cohorts, respectively, reported at least one form of ACE, with higher proportions reporting household mental illness (23% and 52%), sexual abuse (21% and 17%), and psychological abuse (22% and 29%).

**Table 1:** Adverse childhood experiences according to women with and without endometriosis, born in 1973–78 and 1989–95^a^

Adverse childhood experiences	1973–78 cohort (*n* = 3735)			1989–95 cohort (*n* = 4837)		
	Endometriosis		*p*-value	Endometriosis		*p*-value^b^
	Yes (*n* = 590)	No (*n* = 3145)		Yes (*n* = 530)	No (*n* = 4307)	
*Psychological abuse, n (%)*						
Yes No	113 (22.2) 396 (77.8)	494 (17.9) 2265 (82.1)	0.02	143 (28.6) 357 (71.4)	942 (22.8) 3191 (77.2)	0.004
*Physical abuse, n (%)*						
Yes No	59 (11.6) 450 (88.4)	229 (8.3) 2530 (91.7)	0.01	63 (12.6) 437 (87.4)	394 (9.5) 3739 (90.5)	0.03
*Sexual abuse, n (%)*						
Yes No	105 (20.6) 404 (79.4)	437 (15.8) 2322 (84.2)	0.008	84 (16.8) 415 (83.2)	462 (11.2) 3649 (88.8)	< 0.0001
*Household substance abuse, n (%)*						
Yes No	100 (19.7) 409 (80.3)	459 (16.6) 2300 (83.4)	0.09	147 (29.2) 357 (70.8)	947 (22.8) 3203 (77.2)	0.002
*Parental violent treatment, n (%)*						
Yes No	67 (13.2) 442 (86.6)	263 (9.5) 2496 (90.5)	0.01	69 (13.7) 435 (86.3)	477 (11.5) 3673 (88.5)	0.15
*Household mental illness, n (%)*						
Yes No	118 (23.2) 391 (76.8)	490 (17.8) 2269 (82.2)	0.004	260 (51.6) 244 (48.4)	1709 (41.2) 2441 (58.8)	< 0.0001
*Household criminal behaviour, n (%)*						
Yes No	9 (1.8) 500 (98.2)	45 (1.6) 2714 (98.4)	0.82	15 (3.0) 489 (97.0)	77 (1.9) 4073 (98.1)	0.09
*ACEs score (range 0–7)* ^c^						
Median (Q1, Q3)	0 (0, 2)	0 (0, 1)	0.001	1 (0, 2)	1 (0, 2)	< 0.0001
*Childhood traumatic experience, n (%)* ^d^						
Not at all Somewhat Moderately Very/extremely	302 (52.3) 166 (28.8) 63 (10.9) 46 (8.0)	1828 (59.4) 776 (25.2) 260 (8.4) 213 (6.9)	0.01	189 (36.1) 177 (33.8) 79 (15.1) 78 (14.9)	1937 (45.6) 1353 (31.8) 553 (13.0) 406 (9.6)	< 0.0001

^a^Values are frequency (n) and column percentage (%)

^b^p-values from the Pearson chi-square test

^c^The adverse childhood experiences (ACEs) score (range 0–7) was presented as the median (quartiles) using the Mann-Whitney test

^d^Childhood traumatic experience: Due to the low number of responses in the “extremely” category, it was merged with the “very” category for analysis

In the 1973–78 cohort, missing data were observed for each ACE (*n* = 81 with endometriosis; *n* = 386 without) and childhood trauma (*n* = 13 and *n* = 68, respectively). In the 1989–95 cohort, missing ACE data ranged from 26–31 among women with endometriosis and 157–196 among those without. Specifically: psychological abuse (*n* = 30 and *n* = 174), physical abuse (*n* = 30 and *n* = 174), sexual abuse (*n* = 31 and *n* = 196), household substance abuse (*n* = 26 and *n* = 157), parental violent treatment (*n* = 26 and *n* = 157), household mental illness (*n* = 26 and *n* = 157), household criminal behaviour (*n* = 26 and *n* = 157), cumulative ACEs score (*n* = 32 and *n* = 207), and childhood trauma (*n* = 7 and *n* = 58)

Among women with endometriosis, women with ACEs were more likely to report poorer HRQoL than those without such experiences (Supplementary Tables 1 and 2). Across both cohorts, women with ACEs had significantly higher median EHP-30 scores across all domains compared to those without ACEs, except for those who had experienced household criminal behaviour and substance use. In the 1973-78 cohort, exposure to parental violent treatment was linked to significant median differences across all HRQoL domains. However, in the 1989-95 cohort, these differences were not statistically significant, particularly in the domains of emotional well-being and self-image.

Across both cohorts, a high proportion of women grew up in households with lower parental education, difficulty managing on family income, and exposure to parental divorce or separation during childhood (Supplementary Table 3). Significant median differences in cumulative ACE scores were observed by parental education, childhood financial circumstances, and parental divorce or separation, with women from these disadvantaged or disrupted family environments more likely to report higher ACEs.

A dose-response relationship was evident, with higher cumulative ACE exposures and greater levels of childhood trauma (mild to severe) associated with progressively worse HRQoL overall after adjusting for parental socio-demographic factors (Table 2). Across both cohorts, an increase in ACE scores (0–7 points) was associated with poorer outcomes in all EHP-30 domains. Adjusted ORs ranged from 1.29 to 1.41 in the 1973–78 cohort and from 1.22 to 1.27 in the 1989–95 cohort. In the 1973–78 cohort, compared with women reporting no childhood traumatic experiences, those reporting greater trauma had progressively higher odds of being in worse HRQoL categories across all domains; the strongest associations were for perceived social support (OR 4.42, 95% CI 2.36, 8.28), emotional well-being (OR 4.38, 95% CI 2.36, 8.12), and self-image (OR 3.29, 95% CI 1.77, 6.11) among women in the very/extremely traumatic group. Similar patterns were observed in the 1989–95 cohort.

**Table 2:** Associations between adverse childhood experiences and health-related quality of life domains among women with endometriosis

Adverse childhood experiences (ACEs)	Endometriosis health profile-30 domains, adjusted odds ratios (95% CIs)^a^				
	Pain	Control and powerlessness	Emotional wellbeing	Social support	Self-image
*ACEs score (count, 0–7 points)*					
1973–78 cohort (*n* = 590) *P*-trend 1989–95 cohort (*n* = 530) *P*-trend	1.29 (1.14, 1.46) < 0.001 1.27 (1.10, 1.47) 0.001	1.30 (1.15, 1.46) < 0.001 1.23 (1.06, 1.42) 0.005	1.30 (1.15, 1.46) < 0.001 1.23 (1.06, 1.41) 0.006	1.40 (1.24, 1.58) < 0.001 1.25 (1.09, 1.44) 0.002	1.41 (1.24, 1.60) < 0.001 1.22 (1.06, 1.41) 0.007
*Childhood traumatic experience*					
1973–78 cohort (*n* = 590) Not at all Somewhat Moderately Very/extremely P-trend	1.00 1.96 (1.32, 2.90) 3.49 (2.03, 5.98) 3.41 (1.80, 6.46) < 0.001	1.00 1.92 (1.31, 2.82) 3.50 (2.05, 5.98) 3.46 (1.87, 6.40) < 0.001	1.00 1.79 (1.21, 2.63) 2.59 (1.52, 4.40) 4.38 (2.36, 8.12) < 0.001	1.00 2.59 (1.76, 3.83) 4.46 (2.57, 7.74) 4.42 (2.36, 8.28) < 0.001	1.00 1.84 (1.25, 2.71) 2.22 (1.32, 3.75) 3.29 (1.77, 6.11) < 0.001
1989–95 cohort (*n* = 530) Somewhat Moderately Very/extremely P-trend	1.39 (0.86, 2.24) 1.75 (0.97, 3.15) 3.70 (1.95, 7.00) < 0.001	1.33 (0.83, 2.13) 2.20 (1.23, 3.95) 3.37 (1.76, 6.45) < 0.001	1.43 (0.90, 2.28) 2.09 (1.16, 3.77) 5.02 (2.58, 9.79) < 0.001	1.36 (0.84, 2.19) 1.96 (1.09, 3.53) 4.29 (2.21, 8.32) < 0.001	1.55 (0.97, 2.48) 1.91 (1.05, 3.50) 5.38 (2.77, 10.46) < 0.001

^a^Adjusted for parental socio-demographic factors during childhood, such as area of residence, parental education, family income, and parental divorce or separation. P-trend values were obtained by modelling cumulative ACE score (0–7) as a continuous variable and childhood traumatic experience as an ordinal variable in ordinal logistic regression models. Data on cumulative ACEs score were collected through self-reported information from the ALSWH study, while childhood traumatic experiences were sourced from the GELLES study

**Fig. 2:**
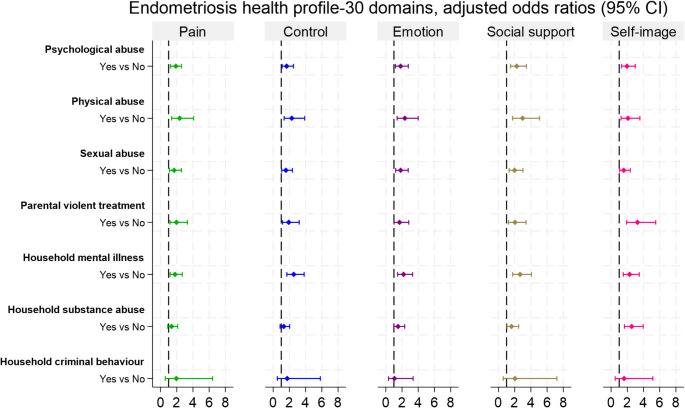
Associations between childhood adversity and health-related quality of life domains among women with endometriosis, 1973–78 cohort (*n* = 590). The odds ratio was adjusted for parental socio-demographic factors during childhood, such as area of residence, parental education, family income, and parental divorce or separation

**Fig. 3:**
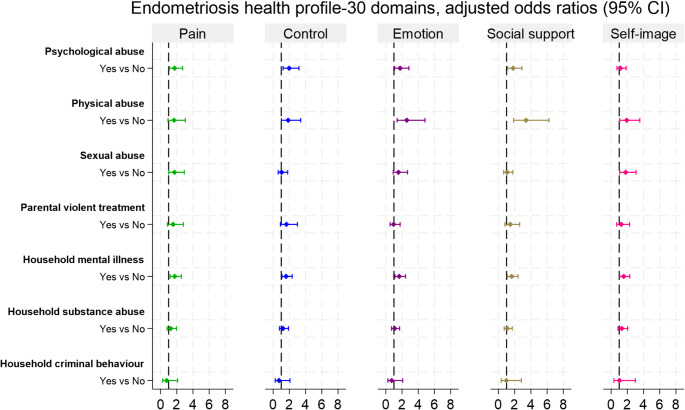
Associations between childhood adversity and health-related quality of life domains among women with endometriosis, 1989–95 cohort (*n* = 530). The odds ratio was adjusted for parental socio-demographic factors during childhood, such as area of residence, parental education, family income, and parental divorce or separation

We examined associations between each type of ACE and HRQoL domains (Figs. 2 and 3). In both cohorts, women with a history of psychological abuse had poorer HRQoL across all domains after adjustment for parental sociodemographic factors. In the 1973–78 cohort, the adjusted odds of being in a worse category ranged from 1.66 to 2.26 across domains, with the largest association for perceived social support (OR 2.26, 95% CI 1.49, 3.45). In the 1989–95 cohort, a similar pattern was observed, except self-image was not statistically significant.

Childhood physical abuse was also significantly associated with poorer overall HRQoL domains in both cohorts, with the strongest association observed in diminished social support. In the 1973–78 cohort, women who experienced physical abuse had two- to threefold higher odds of being in the worse tertiles for pain severity (OR 2.36, 95% CI 1.37, 4.07), control and powerlessness (OR 2.29, 95% CI 1.35, 3.89), emotional distress (OR 2.35 95% CI 1.39, 3.99), social support (OR 2.97, 95% CI 1.74, 5.06), and self-image issues (OR 2.08, 95% CI 1.22, 3.55), compared to those without such experiences. Similar associations were found in the 1989–95 cohort, except for pain, which was not statistically significant.

In the 1973–78 cohort, sexual abuse was associated with a 68% increased likelihood of severe pain (OR 1.68, 95% CI 1.10, 2.57), 83% for emotional distress (OR 1.83, 95% CI 1.21, 2.76), 56% for self-image issues (OR 1.56, 95% CI 1.04, 2.36), and 57% for feelings of powerlessness and lacking control (OR 1.57, 95% CI 1.04, 2.37). The strongest association was with social support needs, nearly doubling the odds (OR 1.99, 95% CI: 1.31, 3.02). In the 1989–95 cohort, sexual abuse was significantly associated with pain severity (OR 1.72, 95% CI 1.01, 2.93) and self-image issues (OR 1.80, 95% CI 1.06, 3.08); however, no significant association was found for the other HRQoL domains.

Childhood exposure to parental violent treatment was associated with a lower HRQoL across all domains in the 1973–78 cohort. This form of adversity was significantly associated with greater pain severity (OR 1.96, 95% CI 1.17, 3.31), heightened feelings of powerlessness and lack of control (OR 1.92, 95% CI 1.15, 3.21), poorer emotional well-being (OR 1.69, 95% CI 1.01, 2.83), diminished social support (OR 2.04, 95% CI 1.23, 3.40), and a notably more negative self-image—the strongest observed association (OR 3.25, 95% CI 1.91, 5.51). However, no significant associations were found in the 1989–95 cohort.

In both cohorts, childhood exposure to household mental illness was strongly associated with a reduced HRQoL across all domains, with the most pronounced association observed in diminished social support. For the 1973-78 cohort, it was associated with higher pain severity (OR 1.78, 95% CI 1.18, 2.68), diminished perceived control/ increased feelings of powerlessness (OR 2.54, 95% CI 1.69, 3.82), poorer emotional well-being (OR 2.19, 95% CI 1.45, 3.31), and lower levels of social support (OR 2.66, 95% CI 1.74, 4.07) and self-image (OR 2.28, 95% CI 1.50, 3.47). Similar findings were observed in the 1989–95 cohort, reinforcing the robustness of these relationships. Household substance abuse was significantly associated with poorer self-image (OR 2.54, 95% CI 1.64, 3.95) and lower social support (OR 1.61, 95% CI 1.05, 2.49) in the 1973–78 cohort; however, no associations were observed in the 1989–95 cohort. Household criminal behaviours showed no significant association with any HRQoL domains in either cohort.

We further analysed each form of ACE with HRQoL outcomes, adjusting for other ACEs, to explore the interplay of multiple childhood adversities and identify which HRQoL domains were most impacted by each specific adversity (Supplementary Tables 4 and 5). In both cohorts, household mental illness remained notably associated with all HRQoL domains, except for pain severity in the 1973-78 cohort, with adjusted ORs ranging from 1.58 to 2.24 in the 1973-78 cohort and from 1.60 to 1.63 in the 1989-95 cohort. Childhood physical abuse had the strongest association with social support in both cohorts, with adjusted ORs 2.08 (95% CI 1.00, 4.35) in the 1973-78 cohort and OR 3.60 (95% CI 1.71, 7.60) in the 1989-95 cohort. In the 1973-78 cohort, sexual abuse was significantly associated with social support (OR 1.68, 95% CI 1.08, 2.59) and emotional well-being (OR 1.58, 95% CI 1.03, 2.43).

A sensitivity analysis using multiple imputation (20 imputations) for missing exposures and confounders showed results consistent with the main analyses, confirming the associations between each ACE and HRQoL domains (Supplementary Tables 6 and 7).

## Discussion

This population-based cohort study demonstrated that women who experienced ACEs were more likely to be diagnosed with endometriosis than those without such experiences. Among women with endometriosis, those with a history of ACEs reported poorer HRQoL across multiple domains compared with women without ACEs. Importantly, a strong dose-response relationship was observed, whereby higher cumulative ACE scores and greater childhood trauma were associated with progressively poorer HRQoL in women with endometriosis, as measured by the validated, disease-specific QoL instrument, EHP-30. Across both cohorts, a history of psychological abuse, physical abuse, and household mental illness was strongly linked to diminished women’s HRQoL across all domains. In the 1973–78 cohort, childhood exposure to sexual abuse and parental violent treatment was associated with lower HRQoL across all domains. However, in the 1989–95 cohort, these estimated associations were generally weaker and less precise, except for sexual abuse, which remained linked to greater pain severity and self-image concerns. No association was found between household criminal behaviours and any HRQoL outcomes.

### Interpretations

A substantial body of evidence indicates that cumulative adversity is a key predictor of long-term health and well-being in the general population [29–31]. However, a more limited number of studies have examined the impact of ACEs on QoL within specific populations. Evidence from cancer survivors showed that multiple ACEs were associated with poorer HRQoL [12], and similar associations were reported among women undergoing hysterectomy for uterine leiomyoma [14] and youth with chronic pain [13]. While our findings align with prior research, our study offers a novel contribution by examining ACEs specifically in women with endometriosis—a population not previously studied in this context. HRQoL was assessed using the EHP-30, the most validated and reliable disease-specific instrument, which captured the multidimensional impact of endometriosis across domains such as pain, emotional well-being, social support, and self-image [18], [19]. The observed dose-response relationship further underscores the cumulative impact of childhood adversity on later-life HRQoL, highlighting the need for targeted screening and intervention strategies.

Psychological abuse was linked to worse pain, greater powerlessness and lack of control, higher emotional distress, and reduced social support among women with endometriosis in both cohorts. However, the findings were attenuated after adjusting for additional ACEs, suggesting a complex interplay between multiple early-life adversities in shaping HRQoL outcomes in women with endometriosis. A prospective cohort study conducted in Canada demonstrated that individuals with a history of emotional abuse reported higher pain interference in daily activities, highlighting the long-term impact of early emotional trauma on pain perception [32].

Childhood physical abuse was also consistently associated with poorer HRQoL across all domains, especially lower social support, even after adjusting for other ACEs. Studies have reported that physical abuse was associated with psychological distress and depressive symptoms in women with chronic pelvic pain [33, 34]. These findings emphasise the long-term impact of childhood physical abuse on HRQoL, particularly regarding social and mental well-being.

Childhood sexual abuse was associated with multiple adverse EHP-30 outcomes, particularly in the 1973–78 cohort, where estimates remained strongly adverse even after adjusting for other ACEs. Our findings underscore the enduring physical, psychological, and social consequences of childhood sexual abuse, aligning with previous studies [35–38]. The most pronounced association was with social support needs, reinforcing existing evidence that childhood sexual abuse can lead to long-term difficulties in forming and maintaining supportive relationships [39, 40]. A meta-analysis of 31 studies involving over 231,000 participants found that individuals with a history of sexual abuse were more likely to report pain conditions, as well as somatic and bowel symptoms [41]. Although the 1989–95 cohort showed similar patterns, the associations were smaller in magnitude and less precise, with clearer indications for pain severity and self-image issues, suggesting potential variations in the impact of sexual abuse across cohorts. These variations may reflect changes in reporting practices, evolving societal awareness, or improvements in support services over time. This study found that the younger cohort (born in 1989-95) reported a lower percentage of sexual abuse, with 12% among women regardless of endometriosis status and 17% among those with endometriosis, compared to the 1973–78 cohort, where the prevalence was 17% and 21%, respectively. This trend indicates a potential decline in sexual abuse over time, consistent with findings from the Australian Child Maltreatment Study, which found lower prevalence among younger cohorts compared with older ones [4]. We found a robust association between childhood exposure to household mental illness and lower HRQoL across all domains in both cohorts, particularly social support. A large body of evidence indicates a strong link between parental mental health issues and the well-being of their children, highlighting the intergenerational effects of mental health disorders [42, 43]. Growing up in a household where mental illness is present can profoundly impact emotional health and social relationships [44, 45]. Importantly, these associations persisted even after accounting for other ACEs, reinforcing the need for targeted interventions to support individuals exposed to early-life familial mental health challenges. Moreover, the pronounced link between household mental illness and diminished social support highlights the crucial role of early family environments in shaping social relationships. Children exposed to parental mental illness often experience inconsistent caregiving, leading to attachment insecurities and long-term difficulties in building and maintaining supportive social networks later in life [46]. Household mental illness more than doubled in the 1989–95 cohort (42% overall, 52% with endometriosis) compared to the 1973–78 cohort (18% and 23%), indicating a rising trend in recent decades. This sharp increase may be driven by societal stressors, economic instability, evolving social structures, and greater awareness and improved diagnosis of mental illness over time, highlighting the need for early detection, improved access to mental health care, and strengthened preventive measures.

Exposure to parental violence was associated with poorer HRQoL in the 1973–78 cohort, with the strongest effect observed on self-image. This association persisted even after adjusting for additional ACEs, suggesting that witnessing parental violence may have a profound impact on self-esteem and body image. These findings align with prior research indicating that early exposure to violence can negatively affect self-esteem [47] and increase vulnerability to mental health issues in later life [48, 49], potentially driven by chronic stress, internalised stigma, and disrupted emotional development. Interestingly, the 1989–95 cohort showed smaller and less precise associations, which might reflect shifts in family dynamics, increased awareness, and improved child protection measures in recent decades. Australia’s initiatives, such as the National Plan to End Violence against Women and Children 2022–2032 [50], along with improved trauma-informed support services, may have helped mitigate the long-term effects of childhood exposure to parental violence on adult QoL.

### Possible explanations

Childhood adversity has been linked to a higher risk of developing endometriosis [51] and a decline in women’s HRQoL. A 2025 case-control study suggests that childhood traumatic experiences may increase the risk of endometriosis independently of shared genetic predisposition, possibly through environmental, psychological, or stress-related pathways [52]. Our previous studies demonstrated that endometriosis is associated with symptoms including dysmenorrhoea, menorrhagia, fatigue, bowel dysfunction, and psychological disorders [9], leading to significant impairments in physical, mental, and social well-being [10]. ACEs may impose an additional burden, further worsening HRQoL in women with endometriosis.

A potential explanation for these associations is the dysregulation of the hypothalamic-pituitary-adrenal axis due to early-life stress, which alters cortisol regulation and contributes to an increased risk of chronic pain, depression, and anxiety – conditions frequently observed in women with endometriosis [53]. Women with ACEs are more susceptible to developing central sensitisation, where pain sensitivity is heightened in response to stimuli, potentially worsening the intensity and duration of endometriosis-related pain [54]. In addition, ACEs are strongly associated with mental health conditions such as depression and anxiety that are reported to worsen endometriosis symptoms and negatively impact overall HRQoL [55], [56].

### Strengths and limitations

This study is the first to examine the relationship between ACEs and HRQoL outcomes in women with endometriosis using the EHP-30 measure. A major strength is the inclusion of two large, population-based cohorts (1973–78 and 1989–95), allowing for the analysis of ACEs across different generational contexts. We assessed individual ACE domains, a cumulative ACE score, and a broader trauma measure to capture different dimensions of childhood adversity and ensure consistency across approaches. The ACE questionnaire is a validated tool for assessing childhood adversity [26], while the EHP-30 has been extensively recognised as a reliable measure of HRQoL, specifically for women with endometriosis [19]. The comprehensive assessment of QoL through the EHP-30 enhances our ability to capture the broad impact of childhood adversity on women with endometriosis. Additionally, self-reported endometriosis diagnoses in the GELLES surveys were validated against ALSWH and linked health records, demonstrating high accuracy [24], consistent with external validation studies (e.g. ) showing that self-reported diagnosis can be a reliable source for case identification [57].

Limitations of this study include the reliance on self-reported ACEs, and the EHP-30 may introduce recall bias. While key parental socio-demographic factors were adjusted for, residual confounding from unmeasured variables (e.g., personality traits and coping mechanisms) cannot be entirely ruled out. Cohort differences in the observed associations suggest that temporal shifts in social, economic, and healthcare environments may have influenced the findings. Given uncertain temporal ordering and complex interplay between ACEs, such adjustments risk overadjustment and may attenuate or distort associations; however, we performed sensitivity analyses with and without these adjustments.

The prevalence of diagnosed endometriosis observed in this study may appear higher than estimates reported globally (~ 10%) [58], where prolonged diagnostic delays remain common. In Australia, prevalence estimates from longitudinal population-based cohorts tend to increase with age and repeated follow-up, reflecting cumulative diagnosis over the life course, alongside recent improvements in awareness and diagnostic practices [59]. The relatively high educational attainment of participants may also have influenced healthcare engagement and diagnosis. In addition, a small proportion of cases (~ 3%) were clinically diagnosed rather than surgically confirmed, which may slightly overestimate prevalence; however, diagnoses were validated against linked health records.

### Clinical implications

Our findings highlight the importance of a trauma-informed approach to endometriosis care, acknowledging the lasting impact of childhood adversity on pain perception, emotional and social well-being. A trauma-informed model emphasises psychological safety, sensitive communication, awareness of potential triggers, and access to mental-health and psychosocial support [60]. While ACEs are associated with poorer health outcomes at the population level, ACE scores have limited utility for individual risk prediction [61, 62]. Any use of ACE assessment should therefore be cautious and integrated within comprehensive, trauma-informed care pathways.

## Conclusion

This study demonstrates that women who experienced ACEs were more likely to be diagnosed with endometriosis than those without such experiences. Among women with endometriosis, a strong dose-response relationship was observed, with higher cumulative ACEs associated with progressively poorer HRQoL. Psychological and physical abuse, along with household mental illness, were consistently linked to lower overall HRQoL domains, while associations with sexual abuse and parental violence varied across cohorts. Our findings underscore the need for trauma-informed care in endometriosis management, integrating routine ACE screening and comprehensive mental health support. The growing burden of household mental illness highlights the need for early intervention. Future research should explore the biological pathways linking ACEs to endometriosis symptoms and HRQoL and their long-term impact on healthcare systems.

## Supplementary Information

Below is the link to the electronic supplementary material.

Supplementary Material 1

## Data Availability

Researchers should contact the principal investigator (GDM) to use the GELLES survey data. Access to linked health data requires approval from Human Research Ethics Committees and Data Custodians.

## Abbreviations

ACEs: Adverse childhood experiences
ALSWH: Australian longitudinal study on women’s health
CIs: Confidence intervals
EHP-30: Endometriosis health profile 30-item
GELLES: Genetic variants, early life exposures, and longitudinal endometriosis symptoms study
HRQoL: Health-related quality of life
ORs: Odds ratios
