# Supplementary material for: Impact of adverse childhood experiences on health-related quality of life in Australian women with endometriosis: a population-based cohort study
Source: Qual Life Res. 2026 Feb 1;35(3):59. doi: 10.1007/s11136-025-04160-1 (PMC12862022; doi:10.1007/s11136-025-04160-1)
Supplement: Supplementary file 1 — Supplementary Material 1 [file 11136_2025_4160_MOESM1_ESM.docx]

**Impact of adverse childhood experiences on health-related quality of life in women with endometriosis: a population-based cohort study**

Dereje G. Gete ^1^, Jenny Doust ^1^, Sally Mortlock ^1^, Jason Abbott ^2^, Gita D. Mishra ^1^

^1^ Australian Women and Girls' Health Research Centre, School of Public Health, The University of Queensland, Brisbane, QLD 4006, Australia

^2^ School of Clinical Medicine, The University of New South Wales, Sydney, NSW 2052, Australia

***Corresponding Author:** Gita D. Mishra

Email: g.mishra@uq.edu.au

ORCID: 0000-0001-9610-5904

**Journal name:** Quality of Life Research

**Supplementary Table 1.** Endometriosis Health Profile-30 domains according to adverse childhood experiences, 1973-78 cohort (n= 590)

| **Adverse childhood experiences** | **Endometriosis Health Profile-30 domains, median (Q1, Q3) ^a^** | | | | |
| --- | --- | --- | --- | --- | --- |
|  | **Pain** | **Control and powerlessness** | **Emotional well-being** | **Social support** | **Self-image** |
| Psychological abuse  Yes  No  p-value ^b^ | 52.3 (40.9, 72.7)  47.7 (27.3, 68.2)  0.002 | 54.2 (37.5, 75.0)  45.8 (20.8, 66.7)  0.002 | 45.8 (33.3, 62.5)  37.5 (20.8, 54.2)  0.0001 | 56.2 (31.2, 75.0)  43.7 (12.5, 62.5)  < 0.0001 | 50.0 (25.0, 66.7)  41.7 (8.3, 50.0)  0.0005 |
| Physical abuse  Yes  No  p-value ^b^ | 56.8 (40.9, 75.0)  47.7 (29.5, 68.2)  0.005 | 62.5 (45.8, 75.0)  45.8 (25.0, 66.7)  0.001 | 50.0 (37.5, 62.5)  37.5 (25.0, 54.2)  0.0003 | 62.5 (37.5, 75.0)  43.7 (18.7, 62.5)  0.0001 | 50.0 (25.0, 66.7)  41.7 (8.3, 83.3)  0.01 |
| Sexual abuse  Yes  No  p-value ^b^ | 52.3 (38.6, 72.7)  47.7 (27.3, 65.9)  0.001 | 50.0 (37.5, 75.0)  45.8 (20.8, 70.8)  0.005 | 45.8 (37.5, 62.5)  37.5 (20.8, 54.2)  < 0.0001 | 56.2 (31.2, 75.0)  43.7 (12.5, 62.5)  0.0002 | 50.0 (33.3, 58.3)  41.7 (8.3, 58.3)  0.03 |
| Household substance abuse  Yes  No  p-value ^b^ | 50.0 (38.6, 70.4)  50.0 (27.3, 68.2)  0.12 | 54.2 (31.2, 70.8)  45.8 (25.0, 70.8)  0.10 | 45.8 (33.3, 56.2)  37.5 (25.0, 54.2)  0.01 | 56.2 (31.2, 75.0)  43.7 (18.7, 62.5)  0.001 | 50.0 (33.3, 66.7)  41.7 (8.3, 50.0)  < 0.0001 |
| Parental violent treatment  Yes  No  p-value ^b^ | 56.8 (40.9, 75.0)  47.7 (27.3, 68.2)  0.002 | 62.5 (45.8, 75.0)  45.8 (20.8, 66.7)  0.002 | 45.8 (29.2, 62.5)  41.7 (25.0, 54.2)  0.01 | 56.2 (37.5, 75.0)  43.7 (18.7, 62.5)  0.0004 | 58.3 (33.3, 75.0)  41.7 (8.3, 50.0)  < 0.0001 |
| Household mental illness  Yes  No  p-value ^b^ | 52.3 (38.6, 70.4)  47.7 (27.3, 68.2)  0.01 | 58.3 (45.8, 75.0)  45.8 (20.8, 66.7)  < 0.0001 | 45.8 (37.5, 62.5)  37.5 (20.8, 54.2)  < 0.0001 | 56.2 (37.5, 75.0)  43.7 (12.5, 62.5)  < 0.0001 | 50.0 (33.3, 66.7)  41.7 (8.3, 50.0)  < 0.0001 |
| Household criminal behaviour  Yes  No  p-value ^b^ | 56.8 (47.7, 70.4)  50.0 (29.5, 68.2)  0.13 | 50.0 (45.8, 70.8)  50.0 (25.0, 70.8)  0.24 | 45.8 (37.5, 50.0)  41.7 (25.0, 54.2)  0.23 | 56.2 (50.0, 75.0)  43.7 (18.7, 62.5)  0.09 | 50.0 (41.6, 58.3)  41.7 (16.7, 58.3)  0.11 |
| Childhood traumatic experiences  Not at all  Somewhat  Moderately  Very/extremely  p-value ^b^ | 40.9 (20.4, 61.4)  52.3 (34.1, 70.4)  56.8 (45.4, 75.0)  59.1 (45.4, 72.7)  0.0001 | 41.7 (16.7, 62.5)  50.0 (29.2, 70.8)  62.5 (45.8, 79.2)  58.3 (45.8, 75.0)  0.0001 | 37.5 (16.7, 50.0)  41.7 (33.3, 54.2)  45.8 (33.3, 58.3)  50 (45.8, 62.5)  0.0001 | 37.5 (12.5, 56.2)  50.0 (25.0, 75.0)  62.5 (31.2, 75.0)  56.2 (37.5, 75.0)  0.0001 | 33.3 (8.3, 50.0)  41.7 (25.0, 66.7)  41.7 (25.0, 66.7)  50.0 (33.3, 75.0)  0.0001 |

^a^ Values are medians (quartiles). ^b^ p-values from the Wilcoxon rank-sum (Mann–Whitney) and Kruskal–Wallis’s test.

The descriptive analysis involved 590 women with endometriosis, with missing data on ACEs for 81 participants and childhood traumatic experience data for 13. For the EHP-30 domains, missing responses ranged from 10 to 27: pain (n = 27), control and powerlessness (n = 17), emotional well-being (n = 15), social support (n = 10), and self-image (n = 14).

**Supplementary Table 2.** Endometriosis Health Profile-30 domains according to adverse childhood experiences, 1989-95 cohort (n= 530)

| **Adverse childhood experiences** | **Endometriosis Health Profile-30 domains, median (Q1, Q3) ^a^** | | | | |
| --- | --- | --- | --- | --- | --- |
|  | **Pain** | **Control and powerlessness** | **Emotional well-being** | **Social support** | **Self-image** |
| Psychological abuse  Yes  No  p-value ^b^ | 70.5 (52.3, 81.8)  63.6 (43.2, 75.0)  0.003 | 70.8 (50.0, 87.5)  62.5 (41.7, 79.2)  0.001 | 58.3 (37.5, 75.0)  50.0 (33.3, 66.7)  0.007 | 62.5 (43.7, 81.2)  56.2 (37.5, 75.0)  0.002 | 66.7 (41.7, 83.3)  58.3 (41.7, 75.0)  0.04 |
| Physical abuse  Yes  No  p-value ^b^ | 72.7 (54.5, 81.8)  65.9 (45.5, 77.3)  0.006 | 75.0 (62.5, 87.5)  62.5 (45.8, 79.2)  0.001 | 66.7 (50.0, 75.0)  50.0 (33.3, 66.7)  < 0.0001 | 68.7 (56.2, 87.5)  56.2 (37.5, 75.0)  < 0.0001 | 75.0 (50.0, 91.7)  58.3 (41.7, 75.0)  0.001 |
| Sexual abuse  Yes  No  p-value ^b^ | 70.5 (52.3, 81.8)  65.9 (45.5, 75.0)  0.05 | 70.8 (45.8, 83.3)  62.5 (45.8, 83.3)  0.31 | 53.3 (41.7, 75.0)  50.0 (33.3, 66.7)  0.03 | 56.2 (50.0, 75.0)  56.2 (37.5, 75.0)  0.19 | 75.0 (50.0, 83.3)  58.3 (41.7, 75.0)  0.009 |
| Household substance abuse  Yes  No  p-value ^b^ | 65.9 (50.0, 81.8)  65.9 (45.5, 77.3)  0.13 | 70.8 (50.0, 79.2)  62.5 (45.8, 83.3)  0.28 | 54.2 (37.5, 66.7)  50.0 (33.3, 66.7)  0.29 | 62.5 (43.7, 75.0)  56.2 (37.5, 75.0)  0.18 | 66.7 (41.7, 83.3)  58.3 (41.7, 75.0)  0.12 |
| Parental violent treatment  Yes  No  p-value ^b^ | 70.5 (52.3, 84.1)  65.9 (45.5, 75.0)  0.05 | 70.8 (50.0, 87.5)  62.5 (45.8, 79.5)  0.06 | 54.2 (37.5, 75.0)  54.2 (33.3, 66.7)  0.33 | 62.5 (43.7, 87.5)  56.2 (37.5, 75.0)  0.03 | 66.7 (41.7, 83.3)  58.3 (41.7, 91.7)  0.19 |
| Household mental illness  Yes  No  p-value ^b^ | 69.3 (50.0, 81.8)  61.4 (43.2, 75.0)  0.007 | 70.8 (50.0, 83.3)  62.5 (41.7, 75.0)  0.01 | 54.2 (37.5, 70.8)  50.0 (33.3, 66.7)  0.009 | 62.5 (43.7, 81.2)  56.2 (37.5, 75.0)  0.02 | 66.7 (41.7, 83.3)  58.3 (33.3, 75.0)  0.02 |
| Household criminal behaviour  Yes  No  p-value ^b^ | 65.9 (45.5, 81.8)  65.9 (47.7, 77.3)  0.93 | 66.7 (33.3, 83.3)  66.7 (45.8, 83.3)  0.87 | 62.5 (29.2, 70.8)  54.2 (37.5, 66.7)  0.63 | 56.2 (31.2, 81.2)  56.2 (37.5, 75.0)  0.91 | 75.0 (50.0, 83.3)  58.3 (41.7, 83.3)  0.41 |
| Childhood traumatic experiences  Not at all  Somewhat  Moderately  Very/extremely  p-value ^b^ | 59.1 (43.2, 75.0)  65.9 (50.0, 75.0)  68.2 (50.0, 79.5)  70.5 (56.8, 81.8)  0.005 | 58.3 (37.5, 75.0)  62.5 (45.8, 79.2)  70.8 (54.2, 83.3)  75.0 (58.3, 83.3)  0.0005 | 45.8 (29.2, 62.5)  50.0 (37.5, 66.7)  58.3 (41.7, 75.0)  66.7 (50.0, 79.2)  0.0001 | 50.0 (31.2, 68.7)  56.2 (37.5, 75.0)  62.5 (43.7, 81.2)  75.0 (56.2, 87.5)  0.0001 | 58.3 (33.3, 75.0)  58.3 (41.7, 75.0)  58.3 (41.7, 83.3)  75.0 (58.3, 91.7)  0.0001 |

^a^ Values are medians (quartiles). ^b^ p-values from the Wilcoxon rank-sum (Mann–Whitney) and Kruskal–Wallis’s test.

This descriptive analysis included 530 women diagnosed with endometriosis. Data on childhood traumatic experiences were missing for 7 participants, while missing data for individual ACEs ranged from 26 to 31: psychological abuse (n = 30), physical abuse (n = 30), sexual abuse (n = 31), household substance use (n = 26), parental violence (n = 26), household mental illness (n = 26), and household criminal behaviour (n = 26). For the EHP-30 domains, missing responses ranged from 6 to 13: pain (n = 12), control and powerlessness (n = 13), emotional well-being (n = 8), social support (n = 8), and self-image (n = 6).

**Supplementary Table 3.** Parental socio-demographic and economic characteristics during childhood by cumulative ACEs ^a^

| **Parental socio-demographic and economic characteristics** | **1973–78 cohort (n= 590)** | | | **1989–95 cohort (n= 530)** | | |
| --- | --- | --- | --- | --- | --- | --- |
|  | The percentage of all women, n (%) | ACEs score (count, 0-7 points), median (Q1, Q3) | p-value ^b^ | The percentage of all women, n (%) | ACEs score (count, 0-7 points) median (Q1, Q3) | p-values ^b^ |
| **Area of residence**  Urban  Rural/remote | 267 (45.2)  307 (52.0) | 0.5 (0, 2)  0 (0, 2) | 0.89 | 303 (57.2)  220 (41.5) | 1 (0, 3)  1 (0, 2) | 0.06 |
| **Parental education**  Up to year 12 or equivalent  Trade/apprenticeship/certificate/diploma  University/higher degree  Don’t know | 348 (60.0)  89 (15.1)  98 (16.6)  43 (7.3) | 1 (0, 2)  1 (0, 2)  0 (0, 2)  0 (0, 2) | 0.25 | 161 (30.4)  95 (17.9)  128 (24.2)  20 (3.8) | 1 (0, 3)  1 (0, 2)  1 (0, 2)  1 (1, 3) | 0.02 |
| **Able to manage on family income**  Impossible/ difficult all the time  Difficult some of the time  Not too bad  Easy  Don’t know | 108 (18.3)  197 (33.4)  181 (30.7)  64 (10.9)  25 (4.2) | 2 (0, 3)  0 (0, 1)  0 (0, 1)  0 (0, 1)  1 (0, 2) | < 0.001 | 52 (9.8)  129 (24.3)  126 (23.8)  84 (15.9)  12 (2.3) | 2 (1, 4)  1 (1, 2)  1 (0, 2)  1 (0, 2)  1.5 (1, 3.5) | < 0.001 |
| **Parental divorce or separation**  Yes  No | 131 (22.2)  444 (75.3) | 1 (0, 3)  0 (0, 2) | < 0.001 | 139 (26.2)  264 (49.8) | 2 (1, 4)  1 (0, 2) | < 0.001 |

In the 1973–78 cohort, missing data were recorded for area of residence (n=16), parental education (n=12), family income (n=15), and parental divorce or separation (n=15), whereas in the 1989–95 cohort, missingness was higher for parental education (n=126), family income (n=127), and parental divorce or separation (n=127), with fewer missing cases for area of residence (n=7).

**Supplementary Table 4.** Association between adverse childhood experiences and health-related quality of life, 1973-78 cohort (n= 590)

| **Adverse childhood experiences (Yes vs No)** | **Endometriosis health profile-30 domains, Adjusted OR (95% CI)** | | | | |
| --- | --- | --- | --- | --- | --- |
|  | **Pain** | **Control and powerlessness** | **Emotional wellbeing** | **Social support** | **Self-image** |
| Psychological abuse: Model 1  Model 2  Model 3 | 1.88 (1.23, 2.87) *  1.89 (1.23, 2.59) *  1.17 (0.65, 2.12) | 1.61 (1.07, 2.42) *  1.66 (1.10, 2.50) *  0.89 (0.50, 1.59) | 1.77 (1.16, 2.69) *  1.82 (1.19, 2.77) *  0.99 (0.54, 1.80) | 2.28 (1.50, 3.46) **  2.26 (1.49, 3.45) **  1.14 (0.62, 2.08) | 1.94 (1.27, 2.95) *  1.95 (1.28, 2.98) *  1.06 (0.58, 1.96) |
| Physical abuse: Model 1  Model 2  Model 3 | 2.36 (1.37, 4.06) *  2.36 (1.37, 4.07) *  1.70 (0.82, 3.53) | 2.26 (1.33, 3.84) *  2.29 (1.35, 3.89) *  1.92 (0.94, 3.92) | 2.35 (1.39, 3.99) *  2.35 (1.39, 3.99) *  1.84 (0.89, 3.80) | 2.99 (1.75, 5.09) **  2.97 (1.74, 5.06) **  2.08 (1.00, 4.35) * | 2.09 (1.22, 3.55) *  2.08 (1.22, 3.55) *  1.11 (0.53, 2.34) |
| Sexual abuse: Model 1  Model 2  Model 3 | 1.69 (1.11, 2.58) *  1.68 (1.10, 2.57) *  1.49 (0.96, 2.31) | 1.58 (1.05, 2.38) *  1.57 (1.04, 2.37) *  1.38 (0.90, 2.11) | 1.83 (1.21, 2.77) *  1.83 (1.21, 2.76) *  1.58 (1.03, 2.43) * | 1.99 (1.32, 3.03) *  1.99 (1.31, 3.02) *  1.68 (1.08, 2.59) * | 1.57 (1.04, 2.37) *  1.56 (1.04, 2.36) *  1.29 (0.84, 1.99) |
| Household substance abuse: Model 1  Model 2  Model 3 | 1.37 (0.89, 2.09)  1.37 (0.89, 2.11)  0.94 (0.57, 1.53) | 1.25 (0.82, 1.92)  1.31 (0.85, 2.02)  0.90 (0.55, 1.47) | 1.46 (0.95, 2.24)  1.51 (0.98, 2.33)  1.08 (0.66, 1.76) | 1.64 (1.07, 2.51) *  1.61 (1.05, 2.49) *  0.98 (0.60, 1.90) | 2.47 (1.60, 3.80) **  2.54 (1.64, 3.95) **  1.85 (1.14, 3.01) * |
| Parental violent treatment: Model 1  Model 2  Model 3 | 1.90 (1.15, 3.16) *  1.96 (1.17, 3.31) *  1.29 (0.71, 2.34) | 1.81 (1.10, 2.99) *  1.92 (1.15, 3.21) *  1.11 (0.61, 2.01) | 1.64 (1.00, 2.73) *  1.69 (1.01, 2.83) *  1.01 (0.56, 1.85) | 2.06 (1.25, 3.42) *  2.04 (1.23, 3.40) *  1.06 (0.59, 1.90) | 3.04 (1.82, 5.10) **  3.25 (1.91, 5.51) **  2.26 (1.24, 4.11) * |
| Household mental illness: Model 1  Model 2  Model 3 | 1.71 (1.14, 2.57) *  1.78 (1.18, 2.68) *  1.42 (0.90, 2.22) | 2.45 (1.64, 3.67) **  2.54 (1.69, 3.82) **  2.24 (1.43, 3.50) ** | 2.12 (1.41, 3.19) **  2.19 (1.45, 3.31) **  1.83 (1.17, 2.85) * | 2.58 (1.69, 3.92) **  2.66 (1.74, 4.07) **  2.10 (1.33, 3.34) * | 2.19 (1.45, 3.31) **  2.28 (1.50, 3.47) **  1.58 (1.01, 2.45) * |
| Household criminal behaviour: Model 1  Model 2  Model 3 | 1.95 (0.60, 6.34)  1.95 (0.59, 6.42)  1.45 (0.43, 4.96) | 1.65 (0.49, 5.54)  1.73 (0.51, 5.82)  1.27 (0.35, 4.54) | 1.05 (0.34, 3.30)  1.06 (0.33, 3.37)  0.70 (0.21, 2.31) | 2.13 (0.61, 7.45)  2.04 (0.58, 7.21)  1.33 (0.37, 4.81) | 1.57 (0.49, 5.00)  1.60 (0.50, 5.15)  0.67 (0.19, 2.35) |

Model 1 adjusted for parental socio-demographic and economic factors during childhood, including area of residence, parental education, and family income. Model 2 additionally adjusted for parental divorce or separation. Model 3 further adjusted for other ACE domains. *p-values < 0.05, **p-values < 0.0001. Women without ACEs (No) were used as the reference group.

The analysis included **590** **women** with endometriosis, with each form of ACE data missing for 81 participants. For the EHP-30 domains, missing responses ranged from 10 to 27: pain (n = 27), control and powerlessness (n = 17), emotional well-being (n = 15), social support (n = 10), and self-image (n = 14).

**Supplementary Table 5.** Association between adverse childhood experiences and health-related quality of life, 1989-95 cohort (n= 530)

| **Adverse childhood experiences (Yes vs No)** | **Endometriosis health profile-30 domains, Adjusted OR (95% CI)** | | | | |
| --- | --- | --- | --- | --- | --- |
|  | **Pain** | **Control and powerlessness** | **Emotional wellbeing** | **Social support** | **Self-image** |
| Psychological abuse: Model 1  Model 2  Model 3 | 1.73 (1.12, 2.65) *  1.73 (1.09, 2.75) *  1.33 (0.75, 2.33) | 1.87 (1.21, 2.91) *  1.97 (1.22, 3.18) *  1.60 (0.89, 2.89) | 1.65 (1.06, 2.57) *  1.76 (1.09, 2.85) *  1.14 (0.64, 2.04) | 1.79 (1.16, 2.77) *  1.81 (1.13, 2.89) *  0.99 (0.56, 1.76) | 1.13 (0.73, 1.76)  1.15 (0.72, 1.85)  0.67 (0.37, 1.20) |
| Physical abuse: Model 1  Model 2  Model 3 | 1.73 (0.97, 3.12)  1.67 (0.92, 3.05)  1.27 (0.62, 2.59) | 1.57 (1.03, 3.38) *  1.85 (1.01, 3.40) *  1.30 (0.62, 2.73) | 2.52 (1.37, 4.63) *  2.59 (1.139, 4.82) *  2.50 (1.17, 5.35) * | 3.42 (1.89, 6.21) **  3.41 (1.86, 6.23) **  3.60 (1.71, 7.60) * | 1.87 (1.03, 3.38) *  1.93 (1.05, 3.54) *  2.31 (1.09, 4.90) * |
| Sexual abuse: Model 1  Model 2  Model 3 | 1.76 (1.04, 2.98) *  1.72 (1.01, 2.93) *  1.52 (0.87, 2.63) | 1.07 (0.63, 1.82)  1.05 (0.62, 1.79)  0.86 (0.49, 1.50) | 1.56 (0.92, 2.64)  1.56 (0.91, 2.67)  1.38 (0.78, 2.42) | 1.09 (0.66, 1.81)  1.07 (0.64, 1.78)  0.85 (0.49, 1.46) | 1.79 (1.05, 3.03) *  1.80 (1.06, 3.08) *  1.66 (0.95, 2.90) |
| Household substance abuse: Model 1  Model 2  Model 3 | 1.31 (0.85, 2.00)  1.26 (0.81, 1.96)  1.11 (0.70, 1.78) | 1.24 (0.81, 1.89)  1.21 (0.78, 1.88)  1.10 (0.70, 1.75) | 1.09 (0.72, 1.67)  1.09 (0.70, 1.70)  1.01 (0.63, 1.61) | 1.14 (0.74, 1.75)  1.09 (0.70, 1.70)  1.02 (0.64, 1.63) | 1.30 (0.85, 1.98)  1.32 (0.85, 2.05)  1.22 (0.76, 1.94) |
| Parental violent treatment: Model 1  Model 2  Model 3 | 1.62 (0.91, 2.88)  1.55 (0.85, 2.82)  1.16 (0.61, 2.19) | 1.65 (0.92, 2.96)  1.63 (0.89, 2.99)  1.32 (0.69, 2.52) | 0.97 (0.54, 1.75)  0.96 (0.53, 1.76)  0.62 (0.32, 1.21) | 1.50 (0.84, 2.69)  1.45 (0.80, 2.62)  0.98 (0.51, 1.86) | 1.24 (0.70, 2.21)  1.26 (0.69, 2.28)  0.96 (0.51, 1.80) |
| Household mental illness: Model 1  Model 2  Model 3 | 1.76 (1.20, 2.59) *  1.74 (1.18, 2.56) *  1.60 (1.07, 2.39) * | 1.60 (1.09, 2.35) *  1.59 (1.08, 2.34) *  1.45 (0.97, 2.17) | 1.64 (1.12, 2.40) *  1.65 (1.12, 2.43) *  1.62 (1.09, 2.43) * | 1.66 (1.13, 2.43) *  1.63 (1.11, 2.41) *  1.63 (1.09, 2.44) * | 1.55 (1.06, 2.27) *  1.56 (1.06, 2.30) *  1.60 (1.07, 2.39) * |
| Household criminal behaviour: Model 1  Model 2  Model 3 | 0.83 (0.30, 2.26)  0.76 (0.27, 2.10)  0.55 (0.19, 1.57) | 0.77 (0.27, 2.18)  0.72 (0.25, 2.07)  0.56 (0.19, 1.67) | 0.73 (0.25, 2.12)  0.72 (0.25, 2.09)  0.59 (0.19, 1.79) | 1.06 (0.38, 3.01)  0.98 (0.34, 2.83)  0.71 (0.23, 2.17) | 1.03 (0.36, 2.96)  1.03 (0.35, 2.99)  0.74 (0.25, 2.21) |

Model 1 adjusted for parental socio-demographic and economic factors during childhood, including area of residence, parental education, and family income. Model 2 additionally adjusted for parental divorce or separation. Model 3 further adjusted for other ACE domains. *p-values < 0.05, **p-values < 0.0001. Women without ACEs (No) were used as the reference group.

The analysis included 530 women diagnosed with endometriosis. Missing data for individual ACEs ranged from 26 to 31: psychological abuse (n = 30), physical abuse (n = 30), sexual abuse (n = 31), household substance use (n = 26), parental violence (n = 26), household mental illness (n = 26), and household criminal behaviour (n = 26). For the EHP-30 domains, missing responses ranged from 6 to 13: pain (n = 12), control and powerlessness (n = 13), emotional well-being (n = 8), social support (n = 8), and self-image (n = 6).

**Supplementary Table 6**. Associations between childhood adversity and health-related quality of life in the 1973-78 cohort after multiple imputations. ^a^

| **Adverse childhood experiences (ACEs) (Yes vs No)** | **Endometriosis health profile-30 domains, Adjusted odds ratios (95% CIs) ^b^** | | | | |
| --- | --- | --- | --- | --- | --- |
|  | **Pain (n= 563)** | **Control and powerlessness (n= 573)** | **Emotional well-being (n= 575)** | **Social support (n= 580)** | **Self-image (n= 576)** |
| Women without ACEs (No) | 1.00 | 1.00 | 1.00 | 1.00 | 1.00 |
| Psychological abuse | 1.90 (1.26, 2.86) * | 1.62 (1.10, 2.41) * | 1.64 (1.10, 2.46) * | 2.27 (1.49, 3.45) ** | 1.88 (1.23, 2.88) * |
| Physical abuse | 2.18 (1.30, 3.67) * | 2.11 (1.27, 3.51) * | 2.11 (1.27, 3.48) * | 2.86 (1.63, 4.99) ** | 1.93 (1.10, 3.37) * |
| Sexual abuse | 1.83 (1.22, 2.76) * | 1.69 (1.13, 2.53) * | 1.79 (1.20, 2.67) * | 2.09 (1.37, 3.19) * | 1.60 (1.04, 2.45) * |
| Household substance abuse | 1.37 (0.91, 2.06) | 1.30 (0.86, 1.97) | 1.37 (0.90, 2.09) | 1.60 (1.04, 2.45) * | 2.46 (1.58, 3.83) ** |
| Parental violent treatment | 2.07 (1.25, 3.42) * | 2.01 (1.21, 3.31) * | 1.63 (1.00, 2.69) * | 2.16 (1.27, 3.67) * | 3.39 (2.01, 5.73) ** |
| Household mental illness | 1.75 (1.19, 2.58) * | 2.42 (1.66, 3.53) ** | 1.95 (1.31, 2.90) ** | 2.51 (1.65, 3.81) ** | 2.19 (1.43, 3.45) ** |
| Household criminal behaviour | 1.81 (0.57, 5.74) | 1.63 (0.43, 6.16) | 1.00 (0.33, 2.93) | 1.95 (0.54, 6.99) | 1.50 (0.49, 4.66) |
| ACEs score (count, 0-7 points) | 1.31 (1.16, 1.49) ** | 1.33 (1.17, 1.50) ** | 1.30 (1.16, 1.46) ** | 1.44 (1.28, 1.62) ** | 1.43 (1.26, 1.62) ** |
| Childhood traumatic experience  Not at all  Somewhat  Moderately  Very/extremely | 1.00  1.90 (1.29, 2.82) *  3.25 (1.89, 5.58) **  3.49 (1.84, 6.60) ** | 1.00  1.96 (1.34, 2.86) *  3.38 (1.98, 5.76) **  3.67 (1.98, 6.80) ** | 1.00  1.79 (1.22, 2.63) *  2.46 (1.45, 4.17) **  4.36 (2.37, 8.02) ** | 1.00  2.56 (1.74, 3.77) **  4.16 (2.41, 7.18) **  4.53 (2.42, 8.47) ** | 1.00  1.84 (1.25, 2.70) *  2.16 (1.29, 3.64) *  3.40 (1.83, 6.29) ** |

^a^ Multiple imputation by chained equations (20 imputations) was performed under the assumption of missing at random to address missing data on exposures (2.2%–13.7%) and confounders (2%–2.7%). Women with no history of adverse childhood experiences **(**ACEs) were used as the reference group. ^b^ Adjusted for parental socio-demographic factors during childhood, such as area of residence, parental education, family income, and parental divorce or separation. *p-values < 0.05, **p-values < 0.0001.

**Supplementary Table 7.** Association between adverse childhood experiences and health-related quality of life in the 1989-95 cohort after multiple imputations. ^a^

| **Adverse childhood experiences (ACEs) (Yes vs No)** | **Endometriosis health profile-30 domains, Adjusted odds ratios (95% CIs) ^b^** | | | | |
| --- | --- | --- | --- | --- | --- |
|  | **Pain (n= 518)** | **Control and powerlessness (n= 517)** | **Emotional well-being (n= 522)** | **Social support (n= 522)** | **Self-image (n= 524)** |
| Women without ACEs (No) | 1.00 | 1.00 | 1.00 | 1.00 | 1.00 |
| Psychological abuse | 1.56 (1.03, 2.36) * | 1.76 (1.15, 2.68) * | 1.61 (1.06, 2.44) * | 1.66 (1.10, 2.50) * | 1.07 (0.71, 1.62) |
| Physical abuse | 1.53 (0.91, 2.57) | 2.17 (1.28, 3.68) * | 2.63 (1.52, 4.54) * | 2.77 (1.61, 4.75) ** | 1.87 (1.10, 3.17) * |
| Sexual abuse | 1.48 (0.93, 2.37) | 1.10 (0.68, 1.76) | 1.42 (0.90, 2.24) | 1.10 (0.71, 1.72) | 1.71 (1.08, 2.71) * |
| Household substance abuse | 1.22 (0.82, 1.81) | 1.16 (0.78, 1.72) | 1.14 (0.76, 1.69) | 1.11 (0.75, 1.65) | 1.15 (0.78, 1.68) |
| Parental violent treatment | 1.47 (0.87, 2.47) | 1.48 (0.88, 2.48) | 1.04 (0.62, 1.72) | 1.40 (0.83, 2.35) | 1.18 (0.71, 1.96) |
| Household mental illness | 1.53 (1.09, 2.16) * | 1.50 (1.06, 2.17) * | 1.45 (1.03, 2.05) * | 1.46 (1.03, 2.05) * | 1.40 (1.01, 1.95) * |
| Household criminal behaviour | 1.08 (0.40, 2.86) | 1.04 (0.37, 2.88) | 1.06 (0.40, 2.87) | 1.12 (0.41, 3.00) | 1.29 (0.49, 3.42) |
| ACEs score (count, 0-7 points) | 1.23 (1.08, 1.40) * | 1.22 (1.08, 1.39) * | 1.20 (1.08, 1.36) * | 1.20 (1.06, 1.36) * | 1.18 (1.03, 1.34) * |
| Childhood traumatic experience  Not at all  Somewhat  Moderately  Very/extremely | 1.00  1.38 (0.91, 2.11)  1.63 (0.96, 2.80)  2.67 (1.54, 4.63) ** | 1.00  1.19 (0.79, 1.80)  1.91 (1.11, 3.28) *  2.46 (1.41, 4.30) ** | 1.00  1.32 (0.87, 1.99)  1.80 (1.05, 3.11) *  3.79 (2.14, 6.70) ** | 1.00  1.16 (0.76, 1.77)  1.74 (1.01, 2.99) *  3.69 (2.08, 6.53) ** | 1.00  1.26 (0.82, 1.90)  1.48 (0.85, 2.57)  3.48 (1.97, 6.16) ** |

^a^ Multiple imputation by chained equations was performed (20 imputations) under the assumption of missing at random to address missing data on exposures (1.3%–6%) and confounders (1.3%–24%). Women with no history of adverse childhood experiences **(**ACEs) were used as the reference group. ^b^ Adjusted for parental socio-demographic factors during childhood, such as area of residence, parental education, family income, and parental divorce or separation. *p-values < 0.05, **p-values < 0.0001.
